# Arterial spin labelling could detect the occlusion of inferior petrosal sinus for cavernous sinus dural arteriovenous fistula

**DOI:** 10.1093/bjrcr/uaae039

**Published:** 2024-10-25

**Authors:** Shota Yoshimura, Yoichi Morofuji, Ryotaro Takahira, Tsuyoshi Izumo, Takayuki Matsuo

**Affiliations:** Department of Neurosurgery, Graduate School of Biomedical Sciences, Nagasaki University, Nagasaki 852-8501, Japan; Department of Neurosurgery, Graduate School of Biomedical Sciences, Nagasaki University, Nagasaki 852-8501, Japan; Department of Neurosurgery, Graduate School of Biomedical Sciences, Nagasaki University, Nagasaki 852-8501, Japan; Department of Neurosurgery, Graduate School of Biomedical Sciences, Nagasaki University, Nagasaki 852-8501, Japan; Department of Neurosurgery, Graduate School of Biomedical Sciences, Nagasaki University, Nagasaki 852-8501, Japan

**Keywords:** arterial spin labelling, digital subtraction angiography, cavernous sinuses, dural arteriovenous fistulas

## Abstract

Several reports indicate that arterial spin labelling (ASL) MRI is useful for the diagnosis, identification of cortical venous reflux, and assessment of therapeutic effect in dural arteriovenous fistula (dAVF). However, there is no reports indicating the utility of ASL in the identification of venous sinus obstruction. We herein report the case of a 72-year-old woman who presented with diplopia and right trigeminal neuralgia due to bilateral cavernous sinus dAVF. Digital subtraction angiography (DSA) showed temporal occlusion of the inferior petrosal sinus (IPS) and ASL indicated hyperintense signal in the IPS. The ASL signal could indicate venous stasis soon after the occlusion based on the serial changes of IPS patency and occlusion observed in the DSA.

## Introduction

Arterial spin labelling (ASL) is an MRI technique that non-invasively assesses the tissue perfusion without exogenous contrast agents. ASL employs endogenous blood as a tracer, allowing the acquisition of perfusion images through the collection of appropriately timed images of arterial blood labelled in the neck. Generally, ASL signal intensity is not detected in the cerebral veins; however, in the presence of an arteriovenous shunt, the ASL signal appears as labelled blood directly migrates from the supplying artery to the draining vein. Although digital subtraction angiography (DSA) is a gold standard for the diagnosis of dural arteriovenous fistula (dAVF) because of high spatial and temporal resolution, DSA is not suitable for frequently repeated follow-up. Several reports on dAVF have highlighted the utility of ASL in diagnosis, evaluation of cortical venous reflux, and assessment of therapeutic outcomes.^1^ ASL is highly sensitivity of 91% and specificity of 96% for the detection of cortical venous drainage.^2^ Herein, we report a case of cavernous sinus dAVF (CS-dAVF) with occlusion of the inferior petrosal sinus (IPS). The hyperintense ASL signal observed in the IPS suggests recent occlusion of the IPS.

## Case presentation

**Figure 1.**
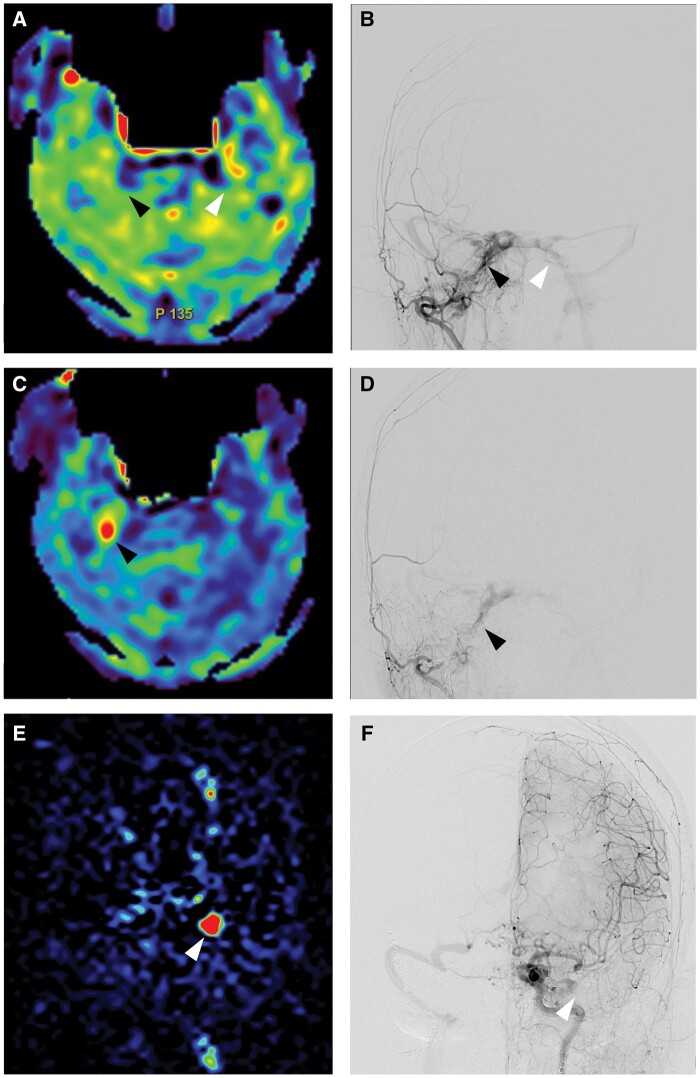
(A) Preoperative arterial spin labelling (ASL) at the level of the IPS (post-labelling delay = 2.0 s) showed the absence of any hyperintense signal around the bilateral IPS (black and white arrowhead). Right (B) anteroposterior external carotid angiography revealed patency of the bilateral IPS (black and white arrowhead). (C) After 3 weeks, ASL showed a hyperintense signal around the right IPS (black arrowhead). (D) Right anteroposterior external carotid angiography indicated occlusion of the right IPS (black arrowhead). (E) One month later, the hyperintense signal around the right IPS disappeared on ASL and a hyperintense signal appeared around the left IPS (white arrowhead). (F) Left anteroposterior internal carotid angiography revealed occlusion of the bilateral IPS (white arrowhead).

A 72-year-old woman presented with headache and nausea and was admitted to another hospital. The patient had no history of trauma or infection. Tinnitus, slight diplopia, and sensory disturbances in the V1 region of the right trigeminal nerve were observed. MR angiography revealed bilateral CS-dAVF. Right carotid angiography showed the presence of a CS-dAVF with a diffuse shunt, which was identified in the anteroinferior component of the cavernous sinus. Multiple arterial feeders supplied the CS-dAVF, including ophthalmic artery, recurrent meningeal artery, ascending pharyngeal artery, internal maxillary artery, middle meningeal artery, and accessory meningeal artery. The bilateral superior and IPSs were patent at this time. Left carotid angiography showed similar feeders and a shunt in the posterosuperior component of the cavernous sinus. The left IPS was patent as a drainer. The patient was discharged after palliative transarterial embolization because of minimal symptoms, and the CS-dAVF had a diffuse shunt point, which may make it difficult to achieve curative treatment even with transvenous embolization (TVE). Initial DSA showed that antegrade flow of the IPS and ASL signal around the IPS were not observed (Figure 1A–B). Three weeks later, an ASL signal was observed around the right IPS, and occlusion of the right IPS was confirmed by DSA (Figure 1C–D). One month later, nausea, dizziness, chemosis, conjunctival hyperaemia, and left abducens nerve paralysis were observed. At that time, the ASL signal around the right IPS completely disappeared and the ASL signal around the left IPS appeared. The left IPS was not observed on DSA (Figure 1E–F). Bilateral IPS occlusion seemed to be the cause of symptom deterioration, and we decided to treat it with TVE due to its aggressive nature and the change in shunt characteristics. The transition observed at the shunt point within each cavernous sinus gradually shifted from a diffuse to a more localized state. Under general anaesthesia, we punctured the femoral arteries and veins bilaterally. Considering the time course and change in the ASL image, we thought it was easy to cross the occluded left IPS. A microcatheter (Excelsior SL-10; Stryker, Kalamazoo, MI, United States) was turned to the origin of the superior ophthalmic vein (SOV), and the tip of the catheter was positioned posterior to the cavernous sinus. However, we were unable to access the right CS through the right IPS. Instead, a microcatheter (Headway 17; MicroVention, Valencia Avenue Tustin, CA, United States) was advanced to the CS via the right facial-SOV. The microcatheter was proceeded from right CS to left CS through inter-CS. The posterior part of the left CS, which was the main shunting point, was obliterated using detachable coils (Target XL 360 soft; Stryker Neurovascular). After confirming the disappearance of the reflux on the left side, we embolized the anterior part of the left CS, interCS, and right cavernous sinus. Final angiography revealed complete occlusion of both dural AVFs. After the operation, her symptoms, except for abducens nerve palsy, improved gradually, and she was transferred to a rehabilitation institute. No postoperative recurrence was confirmed during the 2-year follow-up.

## Discussion

This is the first report of CS-dAVF in which a hyperintense ASL signal in the IPS indicated a recent IPS occlusion. ASL, a non-invasive MRI perfusion technique, is regularly included in standard brain MRI protocols. ASL is a method for measurement of cerebral blood flow by using arterial blood labelled at the neck as an endogenous tracer instead of an exogenous tracer such as contrast media.^3^ The nullification of static tissue signal by background suppression and subtraction of the control from the label image results in a high contrast-to-noise ratio, and only labelled blood produces a signal on the ASL image.^2^ Under normal conditions, 90% of labelled blood is exchanged into brain tissue at the capillary transit and the ASL signal disappears in cerebral veins and sinuses because the T1 decay of labelled water is much shorter than the mean residence time in voxels.^4^ On the other hand, at the arteriovenous shunt in the dAVF, the absence of a capillary bed prevents water extraction and shortens the transit time of labelled water.^5^ These factors induce a hyperintense ASL signal in the venous structure.^6^ The advantage of ASL in dAVF cases is its ability to detect venous reflux or stagnation through an ASL signal, without exogenous contrast agents or radiation exposure. Thus, ASL may be useful for evaluating vascular disorders that can cause dramatic haemodynamic changes, as seen in the present case. On the other hand, S.A. Amukotuwa et al. reported 3 limitations of ASL for dAVFs. First, the technique may not be able to detect drainage veins due to inadequate spatial resolution and delayed post-labelling time. Second, it may depend on the duration of blood flow to traverse the fistula. Third, the ASL may not be sufficiently sensitive to identify low shunt flow.^2^ Several studies have explored ASL findings in acute ischaemic stroke patients. Okazaki et al. reported the utility of ASL in quantitative cerebral blood flow, collateral blood flow, high signal intensity indicative of major vessel occlusion, hyperperfusion post-reperfusion, and crossed cerebellar diaschisis.^7^ High signal intensity in major vessel occlusion indicates labelled blood stasis immediately in front of the cerebral artery embolization site, termed bright vessel appearance (BVA). The high signal intensity observed in the IPS may have resulted from the same underlying mechanism as that of the BVA. In other words, it is considered as an imaging manifestation of blood stasis in proximity to the occlusion site within the venous sinus of dAVF patients. Considering the time course of the ASL and DSA findings, the hyperintense signal reflected stagnant flow proximal to the occlusion site in the IPS shortly after occlusion. During intraoperative catheterization, the catheter was easily guided despite the left IPS occlusion. In contrast, it was difficult to guide the catheter to the right IPS, which had lost the ASL hyperintense signal, suggesting a chronic occlusion of the IPS. This observation may prove valuable for determining the optimal therapeutic intervention approach. The current study has several limitations. First, the same MRI model was not used in this study. Second, an issue arises when evaluating cerebral blood flow with ASL, as varying post labeling delay (PLD) yield disparate images owing to the influence of arterial transit time. In our case, ASL was performed using a post-label delay of 2000 ms, though different ASL images could result from varying parameters. Recently, MR fingerprinting ASL, an imaging technique that does not require PLD, has been investigated and holds promise for applications in diverse pathologies, including cerebrovascular disorders and brain tumours.^8^

In summary, our case showed that the hyperintense signal observed in the IPS on the ASL might be indicative of blood stasis resulting from a recent IPS occlusion. This observation provides valuable information to determine the optimal access route for dAVF treatment.

## Learning points

- Arterial spin labelling (ASL) is useful about the diagnosis, evaluation of cortical venous reflux, and assessment of therapeutic outcomes for dural arteriovenous fistula (dAVF).
- Our case showed that a hyperintense ASL signal observed in the inferior petrosal sinus (IPS) may suggest recent occlusion of the IPS.
- These findings may provide valuable information to determine the optimal access route for dAVF treatment.
